# A Method of Three-Dimensional Micro-Rotational Flow Generation for Biological Applications

**DOI:** 10.3390/mi7080140

**Published:** 2016-08-10

**Authors:** Yaxiaer Yalikun, Yasunari Kanda, Keisuke Morishima

**Affiliations:** 1 Department of Mechanical Engineering, Osaka University, 2-1 Yamadaoka, Suita, Osaka 565-0871, Japan; 2 Laboratory for Integrated Biodevice, Quantitative Biology Center, RIKEN, 1-3 Yamadaoka, Suita, Osaka 565-0871, Japan; 3 Division of Pharmacology, National Institute of Health Sciences, 1-18-1 Kamiyoga, Setagaya, Tokyo 158-8501, Japan; kanda@nihs.go.jp; 4 Global Center for Advanced Medical Engineering and Informatics, Osaka University, 2-2 Yamadaoka, Suita, Osaka 565-0871, Japan

**Keywords:** three-dimensional microfluidic platform, micro-rotational flow, non-contact, open space

## Abstract

We report a convenient method to create a three-dimensional micro-rotational fluidic platform for biological applications in the direction of a vertical plane (out-of-plane) without contact in an open space. Unlike our previous complex fluidic manipulation system, this method uses a micro-rotational flow generated near a single orifice when the solution is pushed from the orifice by using a single pump. The three-dimensional fluidic platform shows good potential for fluidic biological applications such as culturing, stimulating, sorting, and manipulating cells. The pattern and velocity of the micro-rotational flow can be controlled by tuning the parameters such as the flow rate and the liquid-air interface height. We found that bio-objects captured by the micro-rotational flow showed self-rotational motion and orbital motion. Furthermore, the path length and position, velocity, and pattern of the orbital motion of the bio-object could be controlled. To demonstrate our method, we used embryoid body cells. As a result, the orbital motion had a maximum length of 2.4 mm, a maximum acceleration of 0.63 m/s^2^, a frequency of approximately 0.45 Hz, a maximum velocity of 15.4 mm/s, and a maximum rotation speed of 600 rpm. The capability to have bio-objects rotate or move orbitally in three dimensions without contact opens up new research opportunities in three-dimensional microfluidic technology.

## 1. Introduction

With the development of integrated microfluidic technology, the methodology of biological and chemical experiments has become more space saving, more efficient, and requires the use of smaller amounts of reagents and cells. Most of the conventional applications using integrated microfluidic technology are based on a two-dimensional microfluidic system or platform. However, a three-dimensional microfluidic system has been shown to have certain advantages over two-dimensional flow in such fields as tissue engineering and cell differentiation mainly because it requires to create a growth environment that mimics the native tissue as closely as possible [1,2]. Cells cultured in three-dimensional systems enable a larger cell structure and a longer-term incubation owing to the delivery of nutrients to the entire cell structure and tissues [3,4]. However, no use of a three-dimensional culturing system has been reported yet without a scaffold [5], hydrogel [6], specifically designed channel [7,8], additional particle [9] or chamber structure [10]. In the field of cell differentiation, the shear stress generated by the three-dimensional flow can aid in the formation of three-dimensional vascular tubes by increasing the organization of endothelial cells, resulting in a cell response to flow changes according to specific flow parameters [11,12,13] However, in most cases, a stimulation system applies the fluid stimulation on only one side of the cells which means their functionality, phenotype and responses to environmental cues might be altered [14]. In some cases, such as when red blood cells in a vessel are exposed to an alternative flow environment of either a high flow rate (artery, m/s) and a low flow rate (capillary, μm/s) environment, such a large difference in flow rate may induce different phenomena of cell differentiation.

Size-based cell sorting by micro-rotational flow in a closed channel has been previously reported and it is considered to be a high-throughput sorting method [15]. Cells of a specific size can be captured in the micro-rotational flow and the others will flow out. We considered that a three-dimensional micro-rotation flow sorting system in which flow style, acceleration rate, and velocity features are controllable is more efficient and capable of capturing cells in a wider range, and the open environment of sorting makes it easy to retrieve the sorted sample.

For cell manipulation, conventional manipulation systems [16,17,18,19,20] require a complex channel or supportive structure to guide the flow. In addition, fabrication and construction of conventional systems require specific skills in micro-photolithography and use of a complex control system. For example, in our previous work, we reported a three-dimensional microfluidic manipulation system consisting of nine high-specification pumps, a multiple-layer structure microfluidic chip with nine orifices, a control system with a D/A board, a computer and a self-made complex controller [21]. The system functioned by using a self-made control algorithm [22]. However, for practical use in the biological field, a more convenient system requiring no extra chip design, multiple components or complex control system to realize the purposes of capturing, moving and rotating cells is desired.

In this study, we significantly simplified our previous system and we developed a method for creating a three-dimensional micro-rotational fluidic environment by using only a single orifice in a microfluidic chip and a conventional syringe pump. The micro-rotational environment is stable and has a good potential for applications such as culturing, stimulating, sorting, and manipulating cells.

## 2. Methods and Materials

### 2.1. Principles

**Figure 1:**
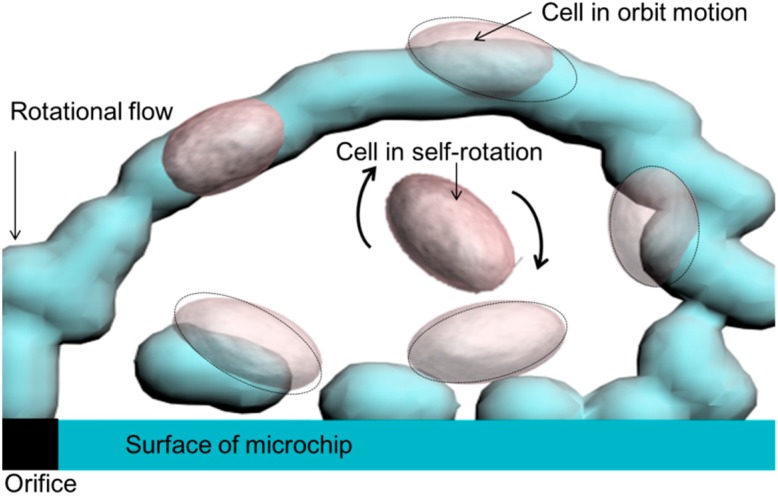
Conceptual illustration of the motion of a cell captured by micro-rotational flow. A cell in the center of the micro-rotational flow performs self-rotational motion. A cell along the rotation flow performs orbital motion.

The concept of our method is based on micro-rotational flow that is generated in an open space. The micro-rotational flow caused by sudden changes in geometries when the fluid flows through has been well documented. Figure 1 shows a schematic representation of cells in a three-dimensional micro-rotational flow. When pushing out solution from a micro-orifice of a microchip to an expansion area, a micro-rotational flow composed of a three-dimensional stable vortex occurs between the air–liquid interface and the surface of the microchip.

In this paper, we consider that formation of micro-rotational flow depends on two main factors: flow velocity and the height of the air–liquid interface. A cell captured by this flow preforms self-rotational motion or orbital motion. By tuning the flow rate, it is potentially possible to rotate the cell that is in a high rate flow, or to achieve a controllable orbital motion over a wide range of sizes and species. Target cells can either be more or less dense than the surrounding medium.

**Figure 2:**
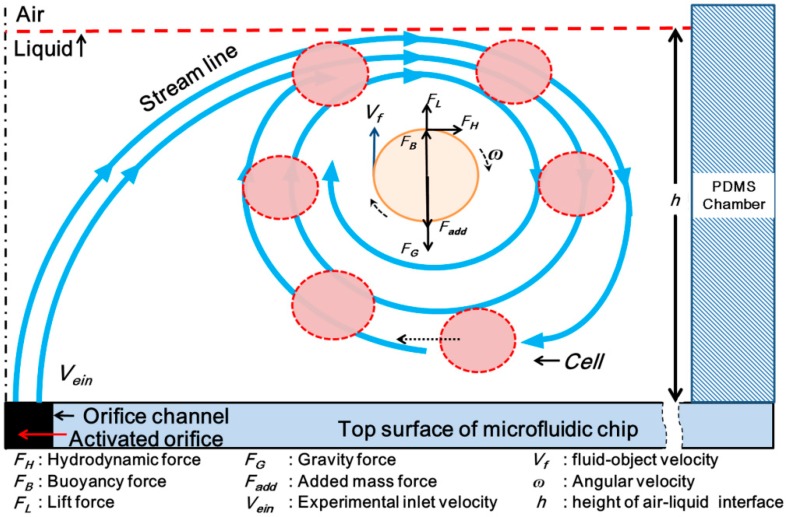
Schematic diagram showing the forces acting on a cell in an assumed equilibrium position of rotational motion and in orbital motion. The cell is captured and moved to the center of the micro-rotational flow in the polydimethylsiloxane (PDMS) chamber. The cell continuously rotates in the zone, where it is exposed to several forces.

To demonstrate this method, estimation of the force necessary for capturing and rotating is required. In our previous research [21], we proved calculation model to estimate driving force for manipulating object and ignored the lift force because object not floating in solution. In this paper, we considered and modified a model of a solid object in vortex flow. When moving uniformly upstream, a rotating sphere in the range of Reynolds number (*R_e_*) < 100, the motion of an individual object obeys Newton’s second law and the force situation is estimated as follows:

(1) $$F_{I}=F_{H}+F_{L}+F_{B}+F_{G}+F_{add}$$

where $F_{I}$ is the total external force exerted on the object in the vortex flow stream, and $F_{H}$,$\;F_{L}$,$F_{B}$, $F_{G}$, and $F_{add}$ are the hydrodynamic force, lift force, buoyancy force, gravity force, and added mass force, respectively, as shown in Figure 2.

The micro-rotational flow exploits a property of fluid itself on its vertical direction. The hydrodynamic force and lift force applied on the fluid–cell interface generate a centripetal force to capture and rotate the cell at the middle of the micro-rotational flow or in length-controlled orbital motion. The hydrodynamic force in the operating area can be expressed by:

(2) $$F_{H}=\frac{1}{2}\rho_{f}C_{D}S{\left(V_{f}-v\right)}^{2}$$

In Equation (2), $\rho_{f}$ is the density of liquid, $C_{D}$ is the hydrodynamic force coefficient, S is the projected section of the cell, $V_{f}$ is the relative velocity between the flow and the rotating object, and v is the object velocity, which was assumed to be 0 m/s in this study. Because the Reynolds number is generally small in a micro-scale device, the hydrodynamic force coefficient can be approximately calculated as follows, when the Reynolds number ($R_{e}$) is 2 × 10^5^ or less. Moreover, in a previous study [23], it was found that the drag coefficient $C_{D}$ (Equation (3)) was not significantly influenced by the rotation of the object.

(3) $$C_{D}\approx \frac{24}{R_{e}}+\frac{6}{1+\sqrt{R_{e}}}+0.4$$

A cell in a shear field experiences a lift force that is perpendicular to the direction of flow. The shear lift originates from the inertia effects in the viscous flow around the cell. The lift force present on a cell can be described as:

(4) $$F_{L}=\frac{1}{2}\rho_{f}C_{L}S{V_{f}}^{2}$$

In Equation (4), $C_{L}$ is the lift force coefficient estimated as 1.4 [24] when $R_{e}$ is 100 or less. The gravity force of the cell in the solution is expressed as:

(5) $$F_{G}=\frac{4}{3}\pi r^{3}g\rho_{o}$$

In Equation (5), r is the radius of the cells, g is gravity force, and $\rho_{o}$ is the density of cells. The buoyancy force of the cell in the solution is expressed as:

(6) $$F_{B}=\frac{4}{3}\pi r^{3}g\rho_{f}$$

In a microfluidic environment, added mass force is the inertia added to an object because an accelerating or decelerating body must move some volume of surrounding fluid as it moves through it:

(7) $$F_{add}=\frac{2}{3}\pi r^{3}g\rho_{f}$$

Continuous rotation of a cell requires stability of the rotation position and rotation speed. To satisfy this requirement, the centripetal force for a rotating cell also needs to balance the hydrodynamic force, lift force, gravity force, buoyancy force, and added mass force. In that situation, an equilibrium situation of a rotating embryoid body (EB) in vertical rotation is established so that the sum of each force present on the cell, $F_{I}$, is zero. Therefore, Equation (1) can be expressed as:

(8) $$(\frac{1}{2}\rho_{f}C_{d}S{\left(V_{f}-v\right)}^{2})+\left(\frac{4}{3}\pi r^{3}g\rho_{f}\right)=\left(-\frac{4}{3}\pi r^{3}g\rho_{o}\right)+\left(\frac{1}{2}\rho_{f}C_{l}S{\left(V_{f}-v\right)}^{2}\right)+\left(\frac{2}{3}\pi r^{3}\rho_{f}\right)$$

This formulation indicates that at the conditions of the specific radius of a cell (diameter of 250 μm), the density of medium solution, velocity of the flow, and lift force coefficient, the bio-object can be rotated. The steady relative velocity between the stream and rotating object $V_{f}$ is calculated to be 0.002217 m/s with a cell rotation speed of 50 rpm. The aim of the calculation is to determine the critical equilibrium velocity condition to estimate the flow rate from the computational fluid dynamics (CFD) software (Ansys, 14.0, Ansys, Inc., Canonsburg, PA, USA) and use it in an experiment. In order to make a bio-object rotate, we calculated the flow rate used in the experiment from Equation (8).

### 2.2. Material

For demonstration of the self-rotational and orbital motions, we focused on iPS (induced pluripotent stem) cells and embryonic stem (ES) cells. After comparing the different uses and culture conditions [25], we chose ES cells. ES cells are obtained from the inner cell mass of the blastocyst of a developing embryo [26]. Because ES cells are pluripotent cells, they can become any type of cell [27]. They are also self-renewable and can be used for tissue regeneration and cellular replacement therapies. Control and manipulation of the numerous differentiation pathways in ES cells have been a topic investigated by numerous researchers [28,29,30,31]. Because an embryoid body (EB) cell is usually large, heavy, and distinct from other bio-objects when exposed to mechanical strain, possibly altering gene expression, most present three-dimensional microfluidic platforms for EBs have been a closed environment; however, retrieving these EBs from the closed environment or cancelling the limitation from around the device structure (such as chamber size limitation) is difficult. Therefore, we demonstrated our method as an option of the three-dimensional microfluidic platform for EB research. 

The EBs were generated using a standard protocol, as previously described [32]. First, mouse R1 ES cells (American Type Culture Collection, Manassas, VA, USA) were thawed on mitomycin C-inactivated mouse embryonic fibroblasts (MEFs; Millipore, Darmstadt, Germany) and cultured on gelatin-coated dishes in ES medium Dulbecco’s modified Eagle’s medium (DMEM; Sigma–Aldrich, St. Louis, MO, USA) supplemented with 10% fetal bovine serum (FBS; Biological Industries, Cromwell, CT, USA), 1000 units/mL leukemia inhibitory factor (LIF; ESGRO, Millipore, Darmstadt, Germany), 1 mM sodium pyruvate (Invitrogen, Thermo Fisher Scientific K.K., Yokohama, Japan), 0.1 mM 2-mercaptoethanol (Sigma–Aldrich), 0.1 mM non-essential amino acids (Invitrogen), 100 units/mL penicillin, and 100 μg/mL streptomycin (Invitrogen)) at 37 °C and 5% CO_2_. After ES cells were dissociated to the single cell suspension using 0.25% Trypsin-EDTA (Gibco, Thermo Fisher Scientific K.K., Yokohama, Japan), one hundred cells were seeded into a non-adherent 96-well U-bottom plate (PrimeSurface; Sumitomo Bakelite, Tokyo, Japan) in DMEM with 10% FBS for 2 days. 

### 2.3. Experimental Device and Rotational and Orbital Motion

**Figure 3:**
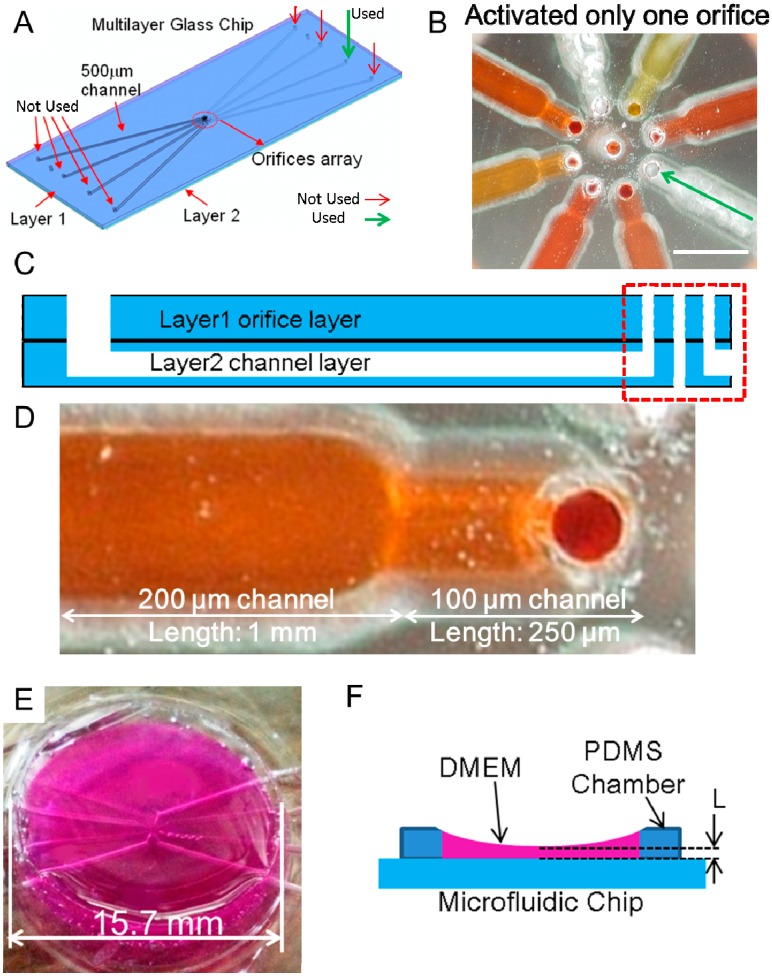
Microfluidic chip used in this study. (**A**) Schematic diagram of the microfluidic chip design. Although there are eight inlets (and channels), we did not use seven of them (indicted by **red** arrows), the inlet and channel we used are indicted by the **green** arrow; (**B**) the orifices in the center area of the microfluidic chip. Each channel was filled with colored solution. The white scale bar is 500 μm; (**C**) schematic diagram of the layer structure of the chip. Layer one had nine orifices, layer two had eight channels; (**D**) there were two channel widths: 100 and 500 μm. The channel depth was 100 μm for all. The diameter of orifice was 100 μm; (**E**) the chamber around the orifices was filled with Dulbecco’s modified Eagle’s medium (DMEM). The designed chamber diameter was 20 mm and the actual size was 15.7 mm); and (**F**) cross-sectional view of the chamber. The air–liquid interface height (*L*) was 1.5 mm.

The microfluidic chip (Figure 3A) described in our previous work was used in this study [21]. It was a two-layer fusion-bonded glass structure chip; the center of the chip is shown in Figure 3B. Layer one (orifice layer) contained eight orifices (orifice diameter, 100 μm) fabricated in a circle (circle diameter, 500 μm) and one orifice fabricated in the middle of the circle by a mechanical drill. In layer two (channel layer), eight channels (each contained three types of channel) were fabricated with a mechanical end mill; one had a width of 500 μm and length of 30 mm, one had a width of 200 μm and length of 1 mm and the last one, a width of 100 μm and length of 250 μm. The two layers were aligned and fusion-bonded to complete the chip assembly. The design details are shown in Figure 3D. In our previous study [21], to achieve the linear motion of an object for a wide range of sizes, and to control the direction of the object, multiple orifices in different positions were used. In addition, at least two orifices and pumps were required to manipulate the object. This kind of control required a complex system design and was not suitable for practical use in biological applications. In this paper, we only activated one orifice and one pump for generation of the micro-rotational flow (Figure 3B). A polydimethylsiloxane (PDMS) chamber was placed on the microfluidic chip, and its topside was open. The actual diameter of the PDMS chamber was 15.7 mm, and its total volume was approximately 600 μL (Figure 3E). The initial height of air-liquid (L) was 1.5 mm (Figure 3F). The experiment was completed before the chamber overflowed, and therefore no outlet port was used.

Experimental images were taken by charge-coupled devices (CCDs, Lu075C, Lumenera, Ottawa, ON, Canada) with zoom lenses (KCM-Z, Tokina, Tokyo, Japan) placed at the top and side of the PDMS chamber (Figure S1), the reflection by the air-liquid interface and the surface wave clearly influence the quality of these images. In the near future, we plan to consider use of a semi-closed or a closed chamber to solve the problems of the reflection and surface wave to achieve better image quality.

The demonstrations of self-rotational motion and orbital motion of the EB were conducted using different processes. In the demonstration of rotational motion, use of an improper flow rate may pull the EBs away from the orifices. Therefore, we initially used the flow rate estimated from the equilibrium position in the CFD simulation. In the demonstration of orbital motion, we used a flow rate that was lower or higher than the one used in the equilibrium position of rotational motion. We observed orbital motion of the EB within a Φ2.5-mm area from the center of the microfluidic chip when the height of the air-liquid interface was *L* = 1.5 mm, as shown in Figure 3F.

### 2.4. CFD Simulation

In order to provide theoretical guidance and understand the field of micro-rotational flow, we conducted the CFD simulation using ANSYS Fluent software (Ansys 14.0, Ansys, Inc.). The goal of the simulation was to reveal the velocity distribution, the micro-rotational flow pattern, and velocity of the particles. A three-dimensional two-phase model was applied to calculate the micro-rotational flow pattern in the PDMS chamber. To simplify the simulation, only flow from the inlet in the liquid domain was considered, and velocity of flow at the orifice $V_{ein}$ was used. The simulation domain we used was 15.7 mm (diameter) × 1.5 mm (depth). The bottom surfaces were set as free convective boundaries at room temperature of 25 °C.

## 3. Results

### 3.1. Experimental Confirmation of the Generation of Micro-Rotational Flow

**Figure 4:**
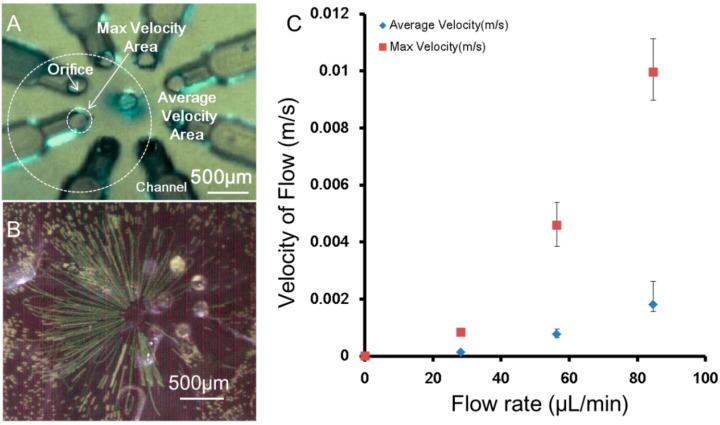
Velocity property of the rotational flow. (**A**) The areas of different velocities; (**B**) visualized area of micro-rotational flow using Fluoro Spheres; and (**C**) velocity comparison between the maximum velocity area and the average velocity area.

Because our system was an open space environment, the velocity of flow from orifice $V_{ein}$ dispersed significantly, and it was not equal to the relative velocity between the stream and rotating object $V_{f}$. Therefore, to experimentally confirm the velocity property of the three-dimensional rotation flow, an experiment was conducted using Fluoro Spheres (20 µm in diameter; Molecular Probes, Invitrogen, Carlsbad, CA, USA). The results showed that the velocity of the flow clearly decreased when being pushed from the orifice. In the maximum velocity area (Figure 4A), the velocity was the same as that calculated from the flow rate; however, in the area of the center of the micro-rotational flow, the velocity was much lower. In addition, flow visualization (Figure 4B) showed the area of the micro-rotational flow was several millimeters from the activated orifice. The velocity measured from the maximum and average velocity areas is shown in Figure 4C. To reach the $V_{f}$ which was calculated to be 0.002217 m/s, a flow rate approximately around 45–85 µL/min was required according to Figure 4C. The flow velocity from the orifice $V_{ein}$ was calculated to be 0.095–0.18 m/s.

### 3.2. Confirmation of the Generation of Micro-Rotational Flow by CFD Simulation

**Figure 5:**
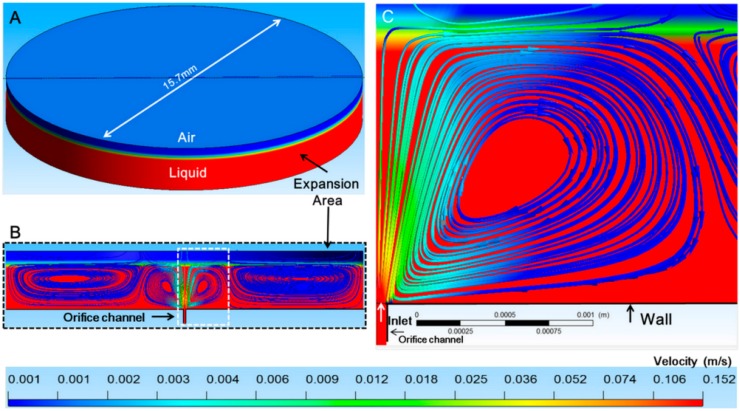
Simulation domain and results. (**A**) The simulation domain contained two phases (air and liquid); (**B**) cross-sectional view of the simulation results; and (**C**) enlarged view of the generated micro-rotational flow.

The cross-sectional view of the simulation results is given in Figure 5B. Figure 5C shows details of the simulated micro-rotational flow in the white dashed line in Figure 5B. The generation of micro-rotational flow with velocity larger than $V_{f}$ was confirmed at a flow rate condition of 71.63 µL/min.

The streamlines indicated that the object in the micro-rotational flow could be exposed to shear velocity. In this situation, a lift force was generated on the object due to the significant difference in velocity and pressure exerted by the stream on opposite sides of the object. When the lift force was powerful enough to balance the hydrodynamic force in the tangential direction and in the direction of the other forces such as the gravity force, added mass force, and buoyancy force, the equilibrium position of the object was obtained. In addition, the velocity of the area near the orifice was clearly faster than the area far from the orifice, and the different flow velocity led to the different rotation speed of the object that was captured.

For the calculated conditions, the diameter of the low velocity core of the micro-rotational flow was from 200 to several hundred micrometers; therefore, the object diameter was set to be several hundred micrometers for the experiment.

### 3.3. Self-Rotational Motion of the EB

**Figure 6:**
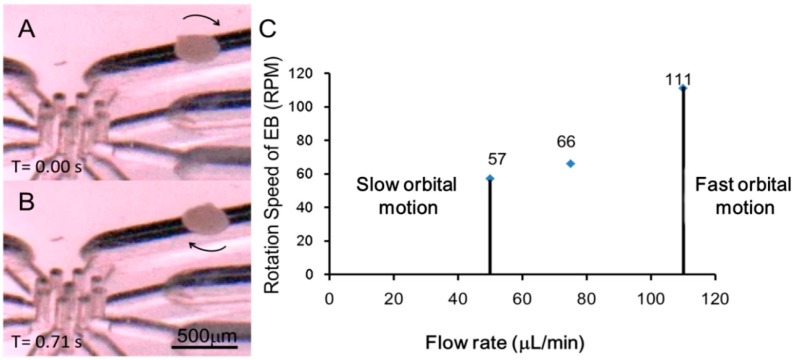
Self-rotational motion of the embryoid body (EB) in DMEM solution. (**A**,**B**) Images from the video footage of EB self-rotation seen from a side and (**C**) the dependence between flow rate and rotation speed.

The results of the self-rotational motion experiment are shown in Figure 6A,B and Video S1. When the rotation of the EB started, the experiment to examine the relationship between the flow rate and rotation speed was conducted. At the estimated flow rate of 71.63 µL/min, the rotation speed of the EB was 66 rpm. The reason for the difference in the rotation speed between the estimated and the experimental results was considered to be due to the density change of the DMEM culture medium caused by evaporation, resulting in a loss of flow rate in the pumping system.

**Figure 7:**
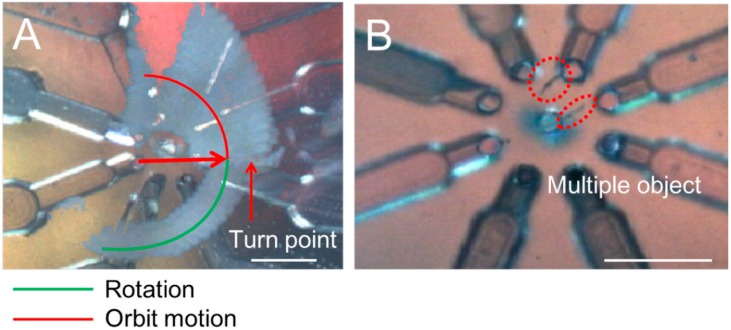
Turning point of rotation. (**A**) The moment that the rotation of the EB became orbital motion; and (**B**) objects of different sizes and species can also be rotated. The scale bar is 500 μm.

The result shown in Figure 6C indicated that the speed of rotation was directly proportional to the flow rate. However, increasing the flow rate to over 109.2 μL/min or decreasing it below 57.4 μL/min induced the self-rotational motion of the EB to become orbital motion. The reason was considered to be that the excessive flow rate caused a significant increase in the lift force, so that the EB was pulled into the stream from the orifice and flowed to the edge of the rotational flow, turning into a slow long-distance orbital motion. In the case of an insufficient flow rate, the lift force was decreased by the change in flow rate, and the force balance was no longer maintained in the equilibrium position. Figure 7A, from Video S2, shows an image at the moment that the rotation shifted to the fast short-distance orbital motion. Moreover, we used two cotton fibers (1.54 g/cm^3^) (diameter of 10 μm, length 200 μm) to demonstrate the system was capable of rotating different objects and also to indicate the possibility that it was capable of manipulating multiple objects. (Figure 7B and Video S3).

### 3.4. Orbital Motion

**Figure 8:**
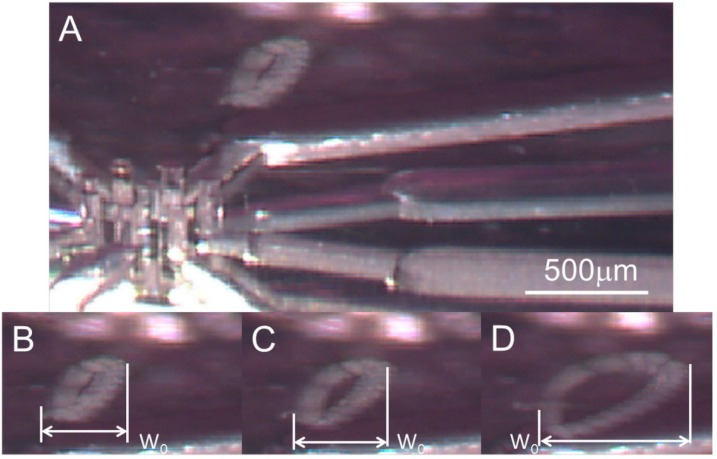
Trajectories of the EB in orbital motion. (**A**) The orbital motion was close to the center area; and (**B**–**D**) images of trajectories obtained under different flow rates of 36.2, 72.5, and 108.7 μL/min.

**Figure 9:**
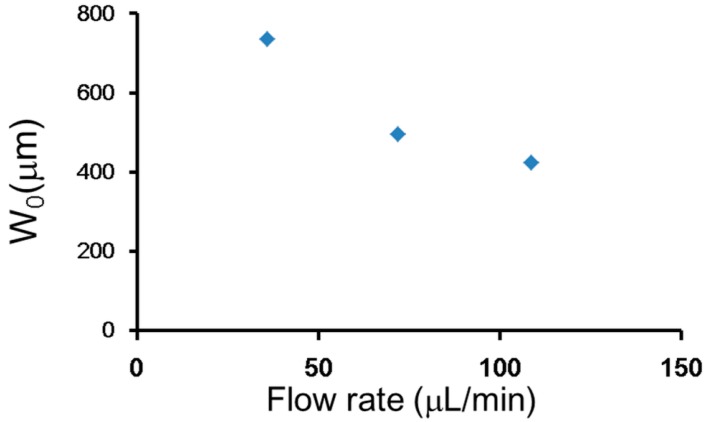
The length on the *x*-axis of trajectories obtained at different flow rates.

The results of the experimental demonstration of the orbital motion of the EB (diameter, 200 μm) are shown in Figure 8 (taken from Video S4). At the estimated flow rates of 36.2 μL/min, 72.5 μL/min, and 108.7 μL/min, the expected distances on the *x*-axis were calculated by the simulation as 419, 837, and 997 μm, respectively. The experimental results are shown in Figure 8B–D, and the distance (in length) is shown in Figure 9; distances of 422.8, 495.8, 734.5 μm were obtained. The reason for the difference between theoretical and experimental values was considered to be the loss of flow rate in the pumping system.

In addition, orbital motion in our system was a variable velocity motion. The acceleration, which is relative to the shear force, is important for the function of the force stimulator for a bio-object. Therefore, the orbital motion experiment of the EB was conducted at the flow rate of 154.9 μL/min, which is the maximum output of our system.

**Figure 10:**
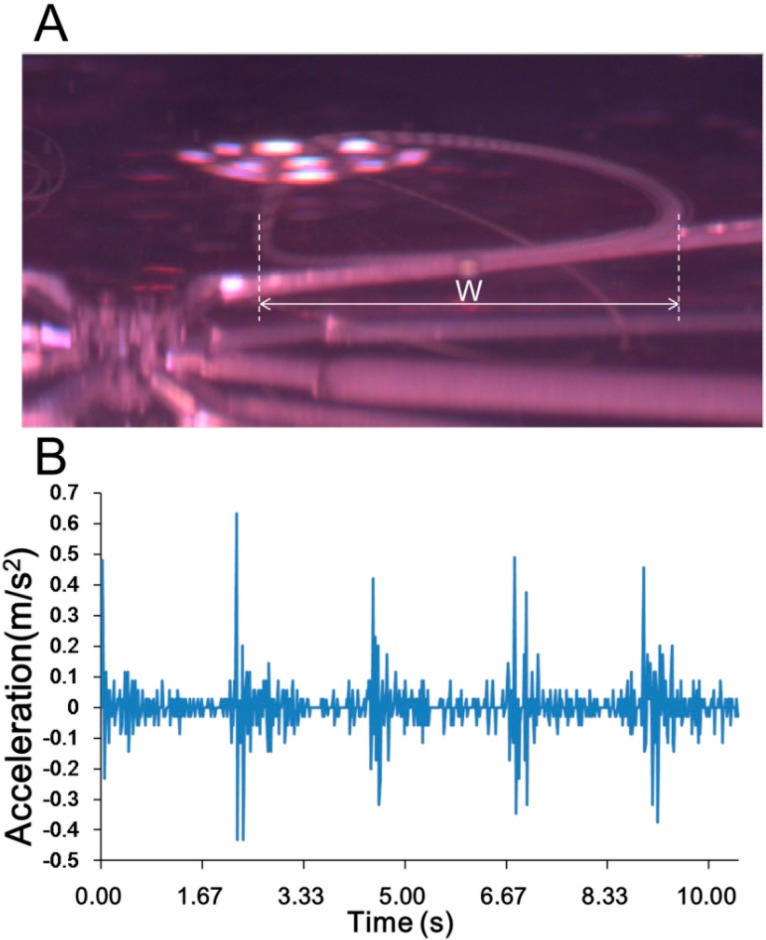
Acceleration of the EB in orbital motion. (**A**) The length on the *x*-axis of trajectories obtained by the imaging process; and (**B**) the acceleration was obtained while the EB was in orbital motion.

**Figure 11:**
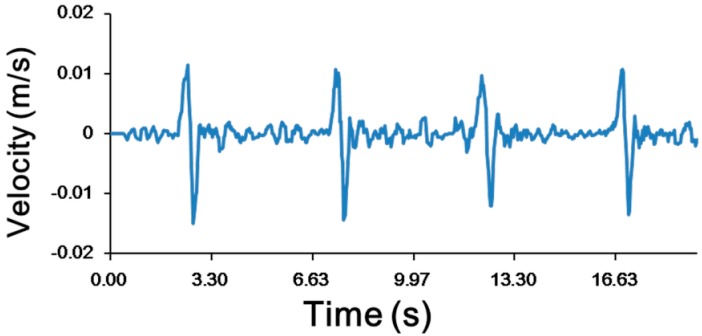
Velocity of the EB in orbital motion. The velocity was obtained from the video recording of the EB in orbital motion.

The trajectory obtained by an imaging process from the recorded video (Video S5) of the orbital motion is shown in Figure 10A, and the relationship between the acceleration in the vertical direction and flow rate is shown in Figure 10B. The maximum acceleration was approximately 0.63 m/s^2^, and the frequency was approximately 0.45 Hz. The maximum speed of the EB was approximately 15.4 mm/s. As shown in Figure 11, cyclic variations in acceleration also induced cyclic variations in the shear force due to velocity. The frequency and acceleration were controllable, which means that the proposed system could function as the force stimulator for the bio-object.

## 4. Discussion

The use of three-dimensional rotational flow enables various possible applications in a microfluidic system. Most of these applications are difficult to obtain with a two-dimensional microfluidic system.

### 4.1. Self-Rotational Motion

The method proposed in this paper is different from other methods based on the use of electric fields, optical forces, surface acoustic wave force, magnetic force, and mechanical tools. We achieved the self-rotational motion of an EB by controlling the micro-rotational flow, and we could determine the relationship between the rotation speed and flow rate. In addition, by regulating the height of the air-liquid interface, the position of rotation was also controllable. Compared with the conventional methods that rotate cells using a complex system, a closed space, and have size and species limitations for the manipulation target, our present method is convenient, conducted in an open space, and allows for a wider size range of the manipulation target (μm–mm). For example, we achieved the rotation speed of 600 rpm of the EB (Video S6) or the orbital motion of the 20 μm diameter EB (Video S7).

### 4.2. Three-Dimensional Culturing Mode

Three-dimensional culturing of a cell or a small amount of cells was possible if the cell had a slow orbital motion or self-rotational motion at a lower rotation speed. Rotating the object cells ensured the supply of nutrition to every part of the cell or the whole cell aggregate. Three-dimensional aggregates of different kinds of cells also can be made and cultured in our system without mechanical agitation. However, unlike other methods using a complex scaffold, microchannel, or micro-chamber, our method is capable of capturing, achieving the short/long-term, fast/slow rotational and orbital motions of an object in a wider size range of the manipulation target (μm–mm) (Video S8).

### 4.3. Three-Dimensional Flow Stimulation Mode: High-Speed Rotation and Acceleration of Flow

This method also showed the potential for practical high-speed rotational motion or orbital motion of a cell. For example, with high-speed rotation, this method can provide the controlled shear stress, which is produced by the steep velocity gradient present in the micro-rotational flow, to the whole bio-object. The effect of shear stress on endothelial cells was reported previously [33]. This method offers a powerful tool capable of presenting shear stress on not only the suspended cells but also on non-suspended bio-objects. Furthermore, unlike other methods that make time-consuming observations of physical changes of cells [34] induced from the free high shear stresses at the bio-object–fluid interface, our system can observe the changes in real time, which may offer new opportunities to study stress-induced signaling events for cells. 

### 4.4. Three-Dimensional Cell Sorting Mode

Chen et al. [35] have reported the two-dimensional orbit motion of cells for different purposes In the present study, the three-dimensional orbital motion of a cell showed a greater rotational flow area than the two-dimensional orbital motion. We found that objects of different sizes and densities performed different motions at the same flow rate (Videos S1 and S9). For example, larger and heavier objects performed self-rotational motion, while smaller and lighter objects performed orbital motion at the same flow rate (Video S1). Thus, by regulating the flow rate, the size of a cell to be rotated in a fixed position can be selected, and the rotating object can be collected easily (on stopping the flow, the object sinks to the surface of the microchip). An orifice is capable of rotating several objects, and increasing the number of orifices can achieve high-throughput three-dimensional cell size sorting.

### 4.5. Three-Dimensional Manipulation

Compared with other conventional methods using hydrodynamic force [16,17,18,19], our method is capable of capturing bio-objects up to several millimeters in size. The capturing process is fast (less than 1 s), and the rotation and moving of the bio-object is simple and effective. Furthermore, the process requires no complex equipment or tools, relying simply on an orifice and a pump.

### 4.6. Comparison with Other Methods used in above Applications

In our evaluated applications, all the operations were conducted using rotational flow. The flow velocity is the most important factor that generates a possible negative influence on the cells. We do not have direct evidence to prove our system has less negative influence because we cannot clarify the reason for the difference between EB cells in a normal situation and after self-rotational motion or orbital motion. However, if we compare the velocity applied on cells in other methods, we can assume our system is bio-friendly and suited for these applications.

As shown in Table 1, in the culturing mode, velocities from 0.127–3 mm/s were previously used; in this paper, we applied velocities up to 2.217 mm/s (slow orbital and rotational motion) on the EBs. The presently applied velocities are clearly lower than those used in previous research, indicating our method does not have a bigger negative influence on cells compared with previous methods. In addition, we observed the EB cells after we had rotated them for 10 min, orbitally moved them for 10 min, and then cultured them for 12 h. For comparison, we prepared EB cells by the same process of culturing them on a glass bottom dish coated with gelatin under the same conditions (Figure S2A,B). There were no significant differences in shape, and no dark zone appeared in the EBs we rotated (Figure S2C,D). 

In the stimulating mode, larger flow velocities increase the effects of fluidic shear stress; however, previous methods were unable to use a flow velocity of more than 3 mm/s due to the risk of EBs being flushed away [30]. In our system, a maximum speed of 15 mm/s can be applied without losing the target EB.

In the sorting and manipulating modes, the flow velocity generating the drag force is normally used; however, in a closed space, velocity-induced pressure effects are not ignorable because our open space system has a lower pressure than a closed system. During the processes, only the force required to cancel the calculated gravity will be applied on the EBs; therefore our method has a smaller pressure effect.

Overall, our system is capable of providing most of the functions that other methods are capable of providing, as well as having a wider range of applied velocities and a smaller pressure effect in an open space.

## 5. Conclusions

We here reported a convenient method of three-dimensional micro-rotational flow generation. This method has potential for such biological applications as culturing, stimulating, sorting, and manipulating different kinds of cells. A controllable micro-rotational flow was generated near an orifice when the solution was pushing from the orifice. The size, velocity, and position of the micro-rotational flow core could be controlled by tuning the parameters such as the flow rate and the liquid-air interface height. A bio-object captured by the micro-rotational flow performed self-rotational motion or orbital motion. In addition, speed and position of rotation, velocity, frequency, and length of orbital motion of cells in the micro-rotational flow could also be controlled. As a typical biological target, EB cells were used to demonstrate our method. We obtained the maximum distance of orbital motion of 2.4 mm, maximum acceleration of 0.63 m/s^2^, frequency of approximately 0.45 Hz, velocity of 15.4 mm/s, and maximum rotation speed of 600 rpm. The capability to have objects rotate or be in orbital motion in three dimensions without contact opens up new research opportunities in three-dimensional microfluidic technology.

## Figures and Tables

**Table 1:** Comparison of environment situation and applied velocity (For embryoid bodies having diameters of 200–300 μm).

Applications	Environment Situation		Applied Velocity (mm/s)	
	Other Methods	This Paper	Other Methods *	This Paper
EB Culturing	Closed	Open	0.127 [28]–3 [29]	Up to 2.217
EB Stimulation	Closed	Open	0.833–3 [30]	Up to 15.4
EB Sorting	Closed	Open	0.383 [31]	Up to 15.4
EB Manipulation	Closed	Open	0.383 [31]	Up to 2.217

* Velocity was calculated from the flow rate and microfluidic chip dimensions that were used in other previous methods

## Supplementary Materials

The following are available online at http://www.mdpi.com/2072-666X/7/8/140/s1. Figure S1: Schematic drawing to show how the image was taken from two sides. There was a CCD camera on the top and another on one side. The side camera was at an angle of 19.5° to the center part of the chip; Figure S2: Images of EBs taken after preparation and culturing—(A) the EBs were prepared from ES cells; (B) after culturing for 48 h, the ES cells were aggregated as EBs on a 96-well U-bottom plate; (C) EBs were removed from the U-bottom plate, and cultured on a gelatin glass bottom dish for 12 h. The image was taken by phase-contrast microscopy; and (D) EBs after rotating for 10 min, orbitally moving them for 10 min, and then culturing them for 12 h. The image was taken by bright field microscopy. Video S1: Self-rotation of EB; Video S2: Rotation speed of an EB at 60 rpm as it changes to orbital motion; Video S3: A single object and two objects were rotated in the micro-rotational flow; Video S4: Short orbital motion of an EB performed in low flow rate; Video S5: Long orbital motion of an EB performed in high flow rate; Video S6: Rotation speed control of an EB; Video S7: Self-rotational motion and orbital motion of an EB (diameter, 20 μm); Video S8: Processes of capturing and performing short and long orbital motions of an EB; Video S9: Different size objects performing orbital motions of different lengths.
