# Supplementary material for: A Method of Three-Dimensional Micro-Rotational Flow Generation for Biological Applications
Source: Micromachines (Basel). 2016 Aug 10;7(8):140. doi: 10.3390/mi7080140 (PMC6190094; doi:10.3390/mi7080140)
Supplement: Supplementary file 1 [file micromachines-07-00140-s001.zip › micromachines-131473-suppl/micromachines-131473-supplementary.pdf]

# Supplementary Materials: A Method of Three-Dimensional Micro-Rotational Flow Generation for Biological Applications

**Yaxiaer Yalikun, Yasunari Kanda and Keisuke Morishima**

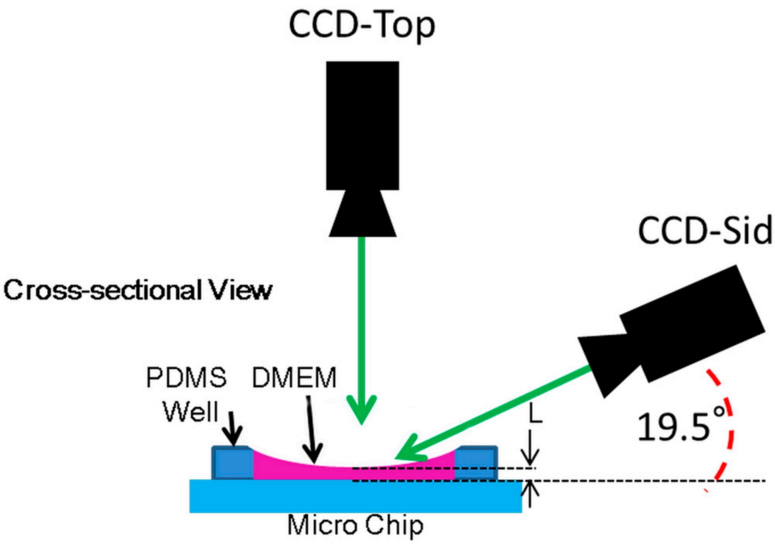


**Figure S1.** Schematic drawing to show how the image was taken from two sides. There was a CCD camera on the top and another on one side. The side camera was at an angle of 19.5° to the center part of the chip.

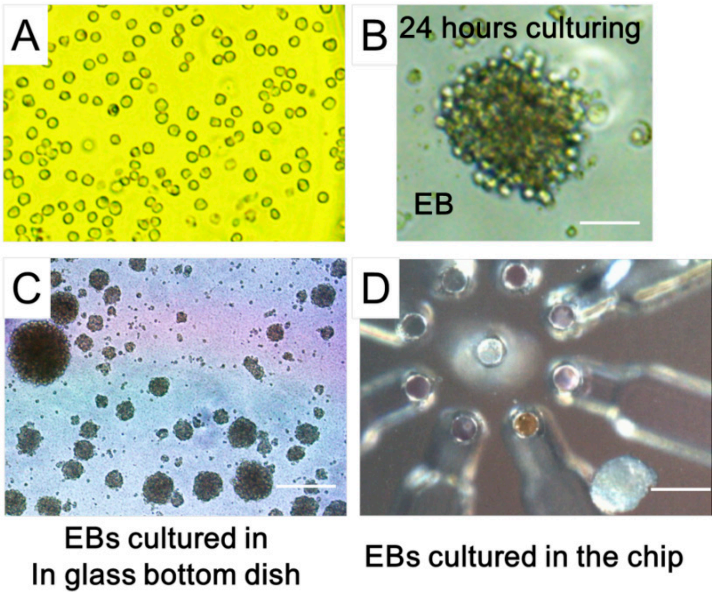


**Figure S2.** Images of EBs taken after preparation and culturing. (**A**) The EBs were prepared from ES cells. (**B**) After culturing for 48 h, the ES cells were aggregated as EBs on a 96-well U-bottom plate. (**C**) EBs were removed from the U-bottom plate, and cultured on a gelatin glass bottom dish for 12 h. The image was taken by phase-contrast microscopy. (**D**) EBs after rotating for 10 min, orbitally moving them for 10 min, then culturing them for 12 h. The image was taken by bright field microscopy.
